# Anaemia requiring red blood cell transfusion is associated with unfavourable 90-day survival in surgical patients with sepsis

**DOI:** 10.1186/s13104-018-3988-z

**Published:** 2018-12-11

**Authors:** Katalin Kristof, Benedikt Büttner, Anna Grimm, Caspar Mewes, Bastian Schmack, Aron Frederik Popov, Michael Ghadimi, Tim Beissbarth, José Hinz, Ingo Bergmann, Ashham Mansur

**Affiliations:** 1 Department of Anesthesiology, University Medical Center, Georg August University, Goettingen, Germany; 2 Department of Cardiac Surgery, University Hospital, Ruprecht Karls University, Heidelberg, Germany; 3 Department of Thoracic and Cardiovascular Surgery, University Medical Center, Goethe University, Frankfurt, Germany; 4 Department of General, Visceral and Pediatric Surgery, University Medical Center, Georg August University, Goettingen, Germany; 5 Department of Medical Statistics, University Medical Center, Georg August University, Goettingen, Germany

**Keywords:** Sepsis, Red blood cell transfusion, Surgical ICU, Mortality, Organ dysfunction, Survival, Organ support, Morbidity

## Abstract

**Objective:**

The mortality associated with sepsis remains unacceptably high, despite modern high-quality intensive care. Based on the results from previous studies, anaemia and its management in patients with sepsis appear to impact outcomes; however, the transfusion policy is still being debated, and the ideal approach may be extremely specific to the individual. This study aimed to investigate the long-term impact of anaemia requiring red blood cell (RBC) transfusion on mortality and disease severity in patients with sepsis. We studied a general surgical intensive care unit (ICU) population, excluding cardiac surgery patients. 435 patients were enrolled in this observational study between 2012 and 2016.

**Results:**

Patients who received RBC transfusion between 28 days before and 28 days after the development of sepsis (n = 302) exhibited a significantly higher 90-day mortality rate (34.1% vs 19.6%; P = 0.004, Kaplan–Meier analysis). This association remained significant after adjusting for confounders in the multivariate Cox regression analysis (hazard ratio 1.68; 95% confidence interval 1.03–2.73; P = 0.035). Patients who received transfusions also showed significantly higher morbidity scores, such as SOFA scores, and ICU lengths of stay compared to patients without transfusions (n = 133). Our results indicate that anaemia and RBC transfusion are associated with unfavourable outcomes in patients with sepsis.

**Electronic supplementary material:**

The online version of this article (10.1186/s13104-018-3988-z) contains supplementary material, which is available to authorized users.

## Introduction

Despite the significant improvements in critical care medicine and intensive research in the field, sepsis and related organ dysfunction remain major causes of mortality, even in the developed world [1]. The pathophysiology of organ failure in patients with life-threatening infections is diverse, including direct cytotoxicity of bacterial toxins, collateral damage through inflammation, and tissue ischemia [2–4]. Since the dysregulation of the microcirculation is common in patients with sepsis [4–7], inadequate perfusion of organs may occur, even if cardiac output and systemic blood pressure are formally in the normal range. Given the reduced density of functioning capillaries in patients with sepsis, theoretical considerations suggest that this patient group might be more sensitive to anaemia, which is indeed very common in septic patients. Anaemia is related to haemolysis, impaired blood clotting with a bleeding tendency and decreased erythrocyte growth [8, 9]. Under these circumstances, the intended benefit of RBC transfusion is to increase oxygen delivery to starving organs; however, the correction of anaemia in this patient group is by far a more complex question in the clinical setting than at the theoretical level [10–14]. Although life-threatening transfusion-related adverse reactions are relatively rare from the classical perspective [15], the administration of blood products represents an allogenic transplantation, which raises several questions. Regarding erythrocytes, the most problematic aspects include transfusion-related immunomodulation [16, 17], impaired rheological properties and reduced oxygen release from stored RBCs [18–20]. Additionally, microparticles originating from stored RBCs exert detrimental effects on homeostasis, including the promotion of coagulation and immunomodulation [21].

Based on two studies revealing the lack of additional benefits of a liberal transfusion regimen from a statistical perspective, recent guidelines recommend a transfusion threshold of 7 g/dL for septic patients without further risk factors, such as myocardial ischemia, severe hypoxemia or acute blood loss [13, 22, 23]. However, a different study concluded that septic patients who received RBC transfusions exhibited reduced 7-day, 28-day and in-hospital mortality after adjustment in the multivariate analysis [24]. These data indicate the heterogeneity of patient groups with sepsis. Indeed, a recent large meta-analysis confirmed that “*the data on RBC transfusions in patients with sepsis are sparse, and the high heterogeneity between studies prevents from drawing any definitive conclusions*” [25]. We focused on a general surgical ICU population with sepsis, excluding cardiac surgery patients due to their particularly high bleeding risk, to investigate the possible associations between anaemia, blood transfusions and outcomes in a homogeneous patient group. Unlike most previous studies, we chose a long (90-day) observation period for mortality as the primary outcome. Because of the immunomodulatory effects of stored allogenic erythrocytes, RBC transfusions as early as 28 days before the beginning of sepsis were considered. The observation period for transfusion was then continued for an additional 28 days after enrolment.

## Main text

### Materials and methods

#### Study population

Surgical patients with sepsis were recruited through the GENOSEP database of the Department of Anesthesiology at the University Medical Center, Goettingen, Germany. This database comprises a prospectively collected cohort of ICU patients with sepsis. All patients treated at the surgical ICUs of the University Medical Center Goettingen between March 2012 and March 2016 were screened for sepsis on a daily basis as described previously [26–28]. Sepsis was defined according to the 2012 standards as a microbiologically or clinically obvious infection with the presence of two or more systemic inflammatory response syndrome (SIRS) criteria, because the new ‘sepsis 3’ definition was not available at the beginning of the study [29]. The exclusion criteria were an age less than 18 years, pregnancy, therapy with immunosuppressive drugs or chemotherapy, human immunodeficiency virus (HIV) infection, chronic heart failure classified as New York Heart Association (NYHA) stage IV, or acute myocardial infarction. Moribund patients were also excluded. No patients enrolled in this study were lost to follow-up. Cardiac surgery patients were not enrolled in this study because of their disproportionally high risk of bleeding.

#### Collection of data

On day 1 (beginning of sepsis and enrolment in the study), the following data were collected: age, gender, pre-existing medical conditions and medications, focus of infection, and SOFA and Acute Physiology and Chronic Health Evaluation (APACHE II) scores. The patients were then followed for 28 days. Over the course of the ICU stay, the following data were collected: vital parameters, routine blood tests, including a blood gas analysis, SOFA score, organ support (renal replacement therapy, mechanical ventilation, and vasopressors), antibiotics used and culture results. Days without organ support were the number of days in the 28-day observation period a patient did not require organ support therapy. We obtained data on the transfusion of erythrocytes in the timeframe of 28 days before and 28 days after the beginning of sepsis. On day 90, the status of our enrolled patients was evaluated.

#### Data analysis

We used Statistica Software (Version 13, StatSoft, Tulsa, Oklahoma, USA) to analyse the data. The significance of the categorical variables was calculated using Pearson’s Chi Square test. Continuous variables were compared using the Mann–Whitney U test. The mortality risk analysis was performed by comparing time-to-event data using the log-rank test. We performed a multivariate Cox regression analysis to exclude the effects of potential confounders and covariates that varied at baseline (e.g., comorbidities) on survival. P values ≤ 0.05 were considered significant. In tables, continuous variables are presented as means ± standard deviations and categorical variables are presented as absolute numbers or percentages.

### Results

#### Baseline characteristics at the time of enrolment

435 Caucasian patients with sepsis who were admitted to the surgical ICUs of the University Medical Center Goettingen were enrolled in this study. Patients in the transfusion group (n = 302) exhibited significantly higher baseline SOFA scores (9.5 ± 3.8 vs. 7.5 ± 3.0; P < 0.001), APACHE II scores (21.6 ± 6.6 vs. 19.0 ± 6.1; P < 0.001) and an increased prevalence of septic shock on day 1 (69% vs. 51%; P < 0.001) compared with patients in the non-transfusion group (n = 133). Patients who received an RBC transfusion required significantly more vasopressors (69% vs. 51%; P < 0.001) and renal replacement therapy (9% vs. 2%; P = 0.004) at baseline. The transfusion group more frequently had a previous medical history of malignant tumours than the transfusion-free group (23% vs. 12%; P = 0.007), whereas a previous history of stroke was rarer in these patients (4% vs. 10%; P = 0.027). No differences in age, gender and comorbidities other than those mentioned above were identified. Although a pulmonary focus followed by an abdominal focus was the most frequent site of infection, patients who received an RBC transfusion experienced a significantly greater incidence of an abdominal focus as site of infection than patients in the transfusion-free group (32% vs. 16%; P = 0.046). All data on baseline characteristics are shown in Table 1.

**Table 1:** Baseline characteristics of surgical patients with sepsis

Parameter	All surgical patients (n = 435)	Patients who received an RBC transfusion (n = 302)	Patients without an RBC transfusion (n = 133)	P value
Age (years)	62 ± 15	63 ± 15	61 ± 15	0.25
Gender (male %)	64	63	65	0.66
BMI (kg/m^2^)	27 ± 6	27 ± 5	28 ± 7	0.93
Septic shock (day 1) [%]	63	69	51	*0.0004*
SOFA score (day 1)	8.9 ± 3.7	9.5 ± 3.8	7.5 ± 3.0	*< 0.001*
APACHE II score (day 1)	20.8 ± 6.5 (n = 430)	21.6 ± 6.6 (n = 300)	19.0 ± 6.1 (n = 130)	*0.0002*
Comorbidities (%)				
Arterial hypertension	52	50	56	0.22
Previous myocardial infarction	5	6	2	0.12
COPD	12	14	8	0.12
Kidney disease	9	10	6	0.22
Diabetes mellitus (insulin-dependent)	9	10	9	0.85
Diabetes mellitus (not insulin-dependent)	8	7	10	0.31
Chronic liver disease	6	7	4	0.24
Malignant tumour	20	23	12	*0.0072*
Stroke	6	4	10	*0.0266*
Site of infection (%)				
Pulmonary	56	52	66	0.14
Abdominal	28	32	16	*0.0457*
Bones or soft tissues	4	5	2	0.45
Surgical wounds	2	2	2	0.54
Urogenital	2	2	3	0.73
Primary bacteraemia	4	5	2	0.58
Other	4	2	7	*0.0130*
Organ support (day 1) [%]				
Mechanical ventilation	85	86	83	0.53
Vasopressors	63	69	51	*0.0004*
Renal replacement therapy	7	9	2	*0.0042*
Medication with statins [%]	20	22	16	0.13

Italic indicates significance of P values (P < 0.05)

#### Mortality analysis

**Fig. 1:**
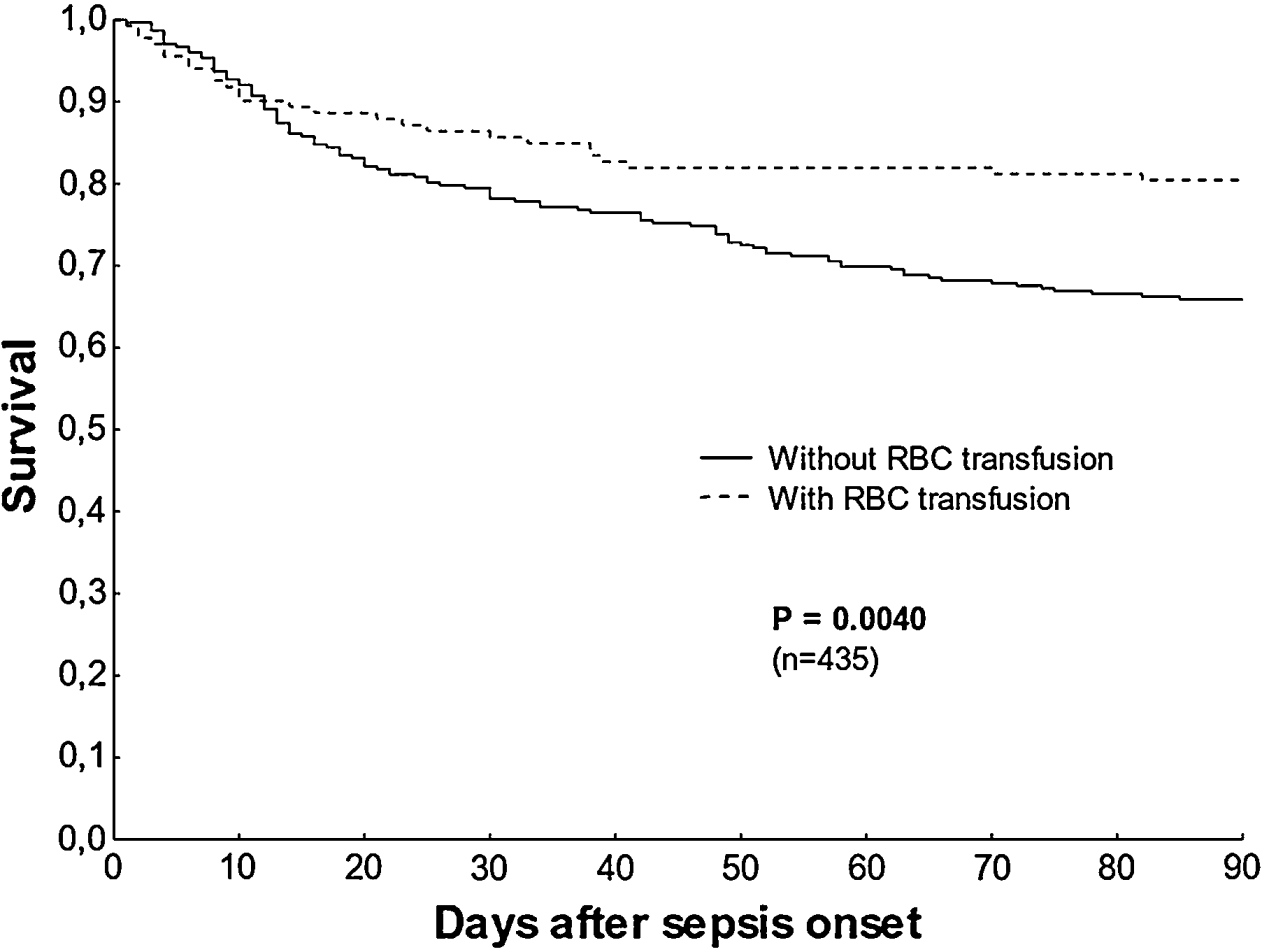
Kaplan–Meier 90-day survival analysis

Patients who received RBC transfusion showed a significantly higher 90-day mortality risk than patients without an RBC transfusion, in the Kaplan–Meier survival analysis (34.1% vs. 19.6%; P = 0.004; Fig. 1). A higher mortality risk among patients in the RBC group was also observed at the 28-day time point, although this difference was not significant (20.2% vs. 13.5%; P = 0.1; Table 2).

**Table 2:** Disease severity of surgical patients with sepsis

Parameter	All surgical patients (n = 435)	Patients who received an RBC transfusion (n = 302)	Patients without an RBC transfusion (n = 133)	P value
SOFA score	6.5 ± 3.2	7.1 ± 3.4	5.2 ± 2.1	*< 0.001*
SOFA-respiratory system	1.8 ± 0.8	1.9- ± 0.8	1.6 ± 0.7	*0.0005*
SOFA-kidneys	0.6 ± 1.0	0.8 ± 1.1	0.2 ± 0.6	*< 0.001*
SOFA-cardiovascular system	1.4 ± 0.9	1.6 ± 0.9	1.1 ± 0.9	*< 0.001*
SOFA-liver	0.3 ± 0.7	0.4 ± 0.7	0.1 ± 0.4	*< 0.001*
SOFA-coagulation	0.3 ± 0.5	0.3 ± 0.6	0.1 ± 0.2	*< 0.001*
SOFA-nervous system	2.0 ± 1.1	2.0 ± 1.1	2.0 ± 1.1	0.86
28-day mortality (%)	18	20.2	13.5	0.10
ICU length of stay (from the beginning of sepsis to discharge)	17 ± 13	20 ± 15	12 ± 7	*< 0.001*
Days without organ support				
Days without mechanical ventilation	5 ± 5	5 ± 6	4 ± 4	0.59
Days without vasopressors	13 ± 8	13 ± 8	12 ± 7	0.19
Days without renal replacement therapy	24 ± 10	14 ± 10	15 ± 8	0.70
Type of infection (%)				
Gram-positive bacteria	83	84	80	0.31
Gram-negative bacteria	69	69	71	0.66
Fungal infection	54	62	35	*<* *0.001*
Viral infection	11	12	10	0.58

Italic indicates significance of P values (P < 0.05)

#### Multivariate analysis

A multivariate Cox regression analysis was performed to adjust for possible effects of different baseline characteristics and potential confounders on 90-day mortality. It included RBC transfusion, potential confounders (age, gender and SOFA and APACHE II scores), and covariates that varied at baseline (septic shock, a medical history of tumours or stroke, abdominal foci and other foci as sites of infection, and vasopressors and renal replacement therapy on day 1). The transfusion of RBCs was independently associated with a higher 90-day mortality (hazard ratio (HR) 1.68; 95% confidence interval (CI) 1.03–2.73; P = 0.035). Further independent risk factors included an age greater than 65 years (HR 1.59; 95% CI 1.08–2.36; P = 0.020) and a higher APACHE II score on day 1 (HR 1.04; 95% CI 1.00–1.08; P = 0.043). Patients with an abdominal focus exhibited a significantly better outcome (HR 0.23; 95% CI 0.06–0.85; P = 0.007). Data are presented in Additional file 1: Table S1.

#### Disease severity

The transfusion group showed a higher average SOFA score during their ICU stay than the group without an RBC transfusion (7.1 ± 3.4 vs. 5.2 ± 2.1; P < 0.001). Regarding the SOFA score, patients transfused with RBCs scored higher on the SOFA-Respiratory system, SOFA-Kidney, SOFA-Cardiovascular system, SOFA-Liver and SOFA-Coagulation subscales. The transfusion group also showed a longer average ICU length of stay (20 ± 15 vs. 12 ± 7 days; P < 0.001) than the non-transfusion group.

The microbiological spectrum of pathogens isolated from cultures of the two patient groups were similar with respect to Gram-positive and Gram-negative pathogens. However, the transfusion group exhibited a significantly higher number of positive fungal cultures (62% vs. 35%; P < 0.001). Disease severity data are illustrated in Table 2.

### Discussion

In this observational study, we investigated the relationship between anaemia requiring an RBC transfusion and 90-day mortality in surgical ICU patients with sepsis. The main finding of the study was that the transfusion of stored allogenic RBCs in a timeframe of 28 days before and after the beginning of sepsis was associated with a significantly higher 90-day mortality risk. This association remained significant after adjustment for confounders.

A causal relationship underlying the higher mortality rate observed in the transfusion group cannot be undisputedly determined from these data. It might be explained by anaemia itself or alternatively by numerous mechanisms by which stored allogenic erythrocytes detrimentally effect homeostasis. Some of these mechanisms, such as impaired rheological properties, reduced oxygen release or the procoagulant effects of RBC-derived microparticles [18–21, 30], develop immediately. Other unfavourable phenomena, particularly transfusion-related immunomodulation (TRIM), evolve over a prolonged period [31]. The mechanisms involved in TRIM are not completely understood, but modulation of cellular immunity is suggested to play a key role [32]. The observation that transfusion decreases the probability of rejection of cardiac and renal allografts [33–35] also supports the hypothesis that stored blood cells exert a lasting modulatory effect on immunity. The increase in the mortality gap between the transfusion and non-transfusion groups during the observation period noted in the present study might support the theory of a probable dominance of some immunological mechanisms responsible for poorer outcomes. The presence of complex immunomodulation associated with RBC transfusion in our patients is also supported by the significantly higher prevalence of positive fungal cultures in the transfusion group.

Septic patients requiring an RBC transfusion showed a more severe presentation of the disease. Upon enrolment, SOFA and APACHE II scores were significantly higher in the transfusion group, and these patients required more vasopressors and renal replacement therapy on day 1. During the observation period, average SOFA scores remained significantly higher in patients who received allogenic RBCs. All SOFA subscores, with the exception of the SOFA-Central nervous system score, were higher in this group.

We also investigated the association between RBC transfusions and pre-existing medical conditions. Patients who received an allogenic blood transfusion were significantly more likely to have some type of malignant tumour in their medical history than patients in the non-transfusion group. This finding is consistent with the observation that anaemia is indeed very common in patients with malignant tumours [36].

### Conclusions

Apart from the mentioned limitations, our findings imply that anaemia requiring the transfusion of stored RBCs in patients with sepsis is associated with higher 90-day mortality rates and disease severity. Further research is needed to determine causal relations.

## Limitations

This observational study has some limitations that need to be highlighted. In our study, we focused on the general surgical ICU population with sepsis; therefore, our results may not be completely applicable to other ICU cohorts. Furthermore, the haemoglobin concentration before the transfusion was not recorded; thus, we were not able to determine whether patients were transfused according to a restrictive or liberal strategy. However, according to the standard operating procedures of our ICUs, patients rarely receive an RBC transfusion if they have a haemoglobin concentration greater than 8 g/dL. Although we observed significant associations between RBC transfusion and sepsis-related mortality and disease severity, our findings do not reveal the causal and biological relations of these observed associations. We postulated that the RBC transfusion exerted a detrimental effect on sepsis outcomes and severity, and patients with a more severe case of sepsis and concomitant anaemia might receive RBC transfusions more frequently. The biological mechanisms underlying our findings must be investigated in further studies.

## Additional file

**Additional file 1: Table S1.** Multivariate Cox regression analysis regarding 90-day survival.

## Abbreviations

APACHE: acute physiology and chronic health evaluation
ICU: intensive care unit
NYHA: New York Heart Association
RBC: red blood cell
SIRS: systemic inflammatory response syndrome
SOFA: sequential organ failure assessment
TRIM: transfusion-related immunomodulation
