# Supplementary material for: Anaemia requiring red blood cell transfusion is associated with unfavourable 90-day survival in surgical patients with sepsis
Source: BMC Res Notes. 2018 Dec 11;11:879. doi: 10.1186/s13104-018-3988-z (PMC6290543; doi:10.1186/s13104-018-3988-z)
Supplement: Supplementary file 1 — Additional file 1: Table S1. Multivariate Cox regression analysis regarding 90-day survival. [file 13104_2018_3988_MOESM1_ESM.docx]

**Additional Material to the manuscript “Anaemia requiring red blood cell transfusion is associated with unfavourable 90-day survival in surgical patients with sepsis”**

| **Parameter** | **Hazard ratio** | **95% confidence interval** | **P value** |
| --- | --- | --- | --- |
| Age over 65 years | 1.59 | 1.08-2.36 | **0.0195** |
| Male gender | 0.96 | 0.66-1.40 | 0.83 |
| SOFA score (Day 1) | 1.09 | 1.00-1.19 | 0.0522 |
| APACHE II score (Day 1) | 1.04 | 1.00-1.08 | **0.0433** |
| **Organ support (Day 1)** | | | |
| Renal replacement therapy | 0.68 | 0.35-1.34 | 0.27 |
| Vasopressors | 1.02 | 0.58-1.81 | 0.94 |
| **Site of infection** | | | |
| Abdominal | 0.23 | 0.06-0.85 | **0.0070** |
| Other | 2.64 | 0.35-20.10 | 0.10 |
| Tumor malignancy in the medical history | 1.63 | 1.07-2.47 | **0.0221** |
| Stroke in the medical history | 1.47 | 0.70-3.07 | 0.31 |
| Medication with statins | 1.13 | 0.73-1.74 | 0.58 |
| **RBC transfusion** | 1.68 | 1.03-2.73 | **0.0354** |

**Table S1: Multivariate Cox regression analysis regarding 90-day survival**
